# Faecal Scent as a Novel Non-Invasive Biomarker to Discriminate between Coeliac Disease and Refractory Coeliac Disease: A Proof of Principle Study

**DOI:** 10.3390/bios9020069

**Published:** 2019-05-27

**Authors:** Maxine D. Rouvroye, Alfian Wicaksono, Sofie Bosch, Edo Savelkoul, James A. Covington, Hanneke Beaumont, Chris J. Mulder, Gerd Bouma, Tim G.J. de Meij, Nanne K.H. de Boer

**Affiliations:** 1 Department of Gastroenterology and Hepatology, Vrije Universiteit Amsterdam, Amsterdam UMC, AG&M research institute, 1081 HZ Amsterdam, The Netherlands; m.rouvroye@vumc.nl (M.D.R.); s.bosch1@vumc.nl (S.B.); edosavelkoul@gmail.com (E.S.); h.beaumont@vumc.nl (H.B.); cjmulder@vumc.nl (C.J.M.); g.bouma@vumc.nl (G.B.); 2 School of Engineering, University of Warwick, Coventry CV4 7AL, UK; A.Wicaksono@warwick.ac.uk (A.W.); J.A.Covington@warwick.ac.uk (J.A.C.); 3 Department of Paediatric Gastroenterology, Vrije Universiteit Amsterdam, Amsterdam UMC, 1081 HZ Amsterdam, The Netherlands; t.demeij@amsterdamumc.nl

**Keywords:** volatile organic compounds, celiac disease, refractory celiac disease, biomarker, non-invasive diagnostics, electronic nose

## Abstract

Currently, the gold standard for diagnosis of coeliac disease (CD) is based on serology and gastroduodenoscopy with histology of duodenal mucosal biopsies. The aim of this study was to evaluate the potential of faecal volatile organic compounds (VOCs) analysis as a novel, non-invasive tool to discriminate between CD in remission in patients on a gluten-free diet (GFD), refractory coeliac disease (RCD) and controls without CD. Patients with an established diagnosis of CD on a GFD, RCD and healthy controls (HC) were instructed to collect a faecal sample. All subjects completed questionnaires on clinical symptoms, lifestyle and dietary information. Faecal VOCs were measured using gas chromatography-ion mobility spectrometry. A total of 13 CD, 7 RCD and 10 HC were included. A significant difference in VOC profiles between CD and RCD patients (area under the curve (AUC) ± 95% CI: 0.91 (0.79–1) *p* = 0.000) and between CD and HC (AUC ± 95% CI: 0.71 (0.51–0.91) *p* = 0.0254) was observed. We found no significant differences between faecal VOC patterns of HC and RCD. Based on faecal VOCs, CD could be discriminated from RCD and HC. This implies that faecal VOC analysis may hold potential as a novel non-invasive biomarker for RCD. Future studies should encompass a larger cohort to further investigate and validate this prior to application in clinical practice.

## 1. Introduction

Coeliac disease (CD) is a chronic, immune-mediated enteropathy triggered by the ingestion of gluten in genetically predisposed individuals, with an estimated prevalence of 1% in Europe [1,2,3]. Only 37% of all diagnosed CD patients portray classical symptoms such as diarrhoea, weight loss and abdominal pain. Most CD patients are asymptomatic or present with extra-intestinal symptoms [4,5,6].

The current gold standard to diagnose CD consists of serological screening for circulating transglutaminase 2 antibodies (TGA2) [7]. Confirmation of diagnosis is required by means of histological examination of duodenal biopsies, which are scored for villous atrophy, crypt hyperplasia and intraepithelial lymphocytes (IEL) [8]. Treatment consists of life-long adherence to a strict gluten-free diet (GFD) [9]. Despite abstaining from gluten, a small minority (0.83 per 10,000 CD patients per year in The Netherlands) develops refractory coeliac disease (RCD) with persistent or recurrent villous atrophy [10]. This is often accompanied by severe diarrhoea, weight loss and malabsorption [11]. Refractory coeliac disease is subdivided in two types. A distinction is made based on histology; whereas RCD I has a benign population of IELs and generally improves after additional treatment, RCD II is characterized by abnormal or clonal IELs and has a poor prognosis with an increased risk for the development of enteropathy associated T cell lymphoma (EATL) [12]. The diagnosis of RCD is challenging and does rely on flow cytometric analysis of freshly isolated duodenal lymphocytes. Since this diagnostic modality is not widely available there is a great need for non-invasive biomarker(s) [13,14].

Analysis of volatile organic compounds (VOCs) is an emerging technique in metabolomics. These carbon compounds are organic chemicals with a high vapour pressure, detectable in faeces, urine, breath, skin and saliva [15]. In the past decades, the use of an electronic nose (a mobile device consisting of chemical sensors coupled with pattern recognition software) has taken flight, and has proven its potential in the detection of urinary tract infections, prostate cancer and lung cancer among others [16,17,18,19]. Gas chromatography (GC) merged with an ion mobility spectrometer (IMS) is a technique that enables the assessment of specific VOC composition, allowing for more sensitive and more specific measurement [20,21]. The potential of VOC analysis as a non-invasive biomarker for the detection of various gastrointestinal diseases has been demonstrated previously [22,23,24,25,26]. Faecal VOCs are responsible for the majority of odours and are believed to largely reflect the composition of the gut microbiota [19]. These characteristics make the use of faecal VOCs as a biomarker for intestinal disease appealing. Faecal VOC profiles can either be obtained from faecal samples or rectal swabs. However, differences in VOC profiles have previously been observed between swab derived and stool sample derived VOCs as the faecal swab itself might influence the VOC [27,28]. Stool samples can be obtained and stored frozen by the subjects until transport and analysis without altering the composition as long as the sample is kept in cool conditions [29]. Differences between the faecal microbiota and VOC profiles of untreated paediatric CD patients and healthy controls have already been demonstrated [30]. Therefore, we hypothesize that analysis of faecal VOCs by means of GC-IMS may also be a useful technique for the differentiation of RCD from CD. The aim of this study was to assess the potential of VOCs from faecal samples as a biomarker specifically for RCD II.

## 2. Materials and Methods

### 2.1. Study Design

In this explorative case-control study, patients were recruited from the Coeliac Centre Amsterdam (Amsterdam University Medical Centre, location Vrije Universiteit Medical Centre, Amsterdam, The Netherlands). Patients who underwent a gastroduodenoscopy between October 2015 and November 2017 were eligible to participate. We included three groups; CD, RCD type II, and a non-coeliac control group that also underwent a gastroduodenoscopy. Inclusion criteria for all groups were adulthood (≥18 years), signed informed consent and comprehension of the Dutch language. Patients were eligible for the CD group if they had a positive tTGA titre (>7.0 U/mL) combined with a Marsh classification >2 of the duodenal biopsies upon diagnosis and if they had followed a strict GFD for ≥2 years [8]. Exclusion criteria were recently (<3 months) elevated levels of tTGA or recently proven villous atrophy. Refractory coeliac disease type II was defined as persisting malabsorption and villous atrophy after one year on a strict GFD ascertained by a dietician, with a clonal expansion of aberrant IELs, regardless of tTGA levels. An endoscopic control group of patients with minor abdominal complaints, scheduled to undergo a gastroduodenoscopy was used as a background population. Patients were eligible to participate as a non-coeliac control, referred to as healthy controls (HC), if no significant mucosal abnormalities were detected at gastroduodenoscopy and no abnormalities were observed on histological examination of biopsies, if taken. Controls were excluded if they abstained from dietary gluten or if they had other significant bowel diseases of possible influence on the VOC profile (e.g., inflammatory bowel disease or colorectal cancer) [24,31].

Patient demographics, medication, gastroduodenoscopy and biopsy reports were obtained from electronic patient records. Information on dietary intake, weight and height, stool consistency based on the Bristol stool chart, smoking status and medication use were collected through a questionnaire.

### 2.2. Sample Collection

Patients included in this study were asked to collect a faecal sample (Stuhlgefäß 10 mL, Frickenhausen, Germany) prior to their scheduled visit. Participants kept the sample in the freezer at home within one hour following collection, and transported this sample to the hospital in a cooled condition using ice packs and/or ice cubes on the day of their visit.

### 2.3. Sample Preparation

From the original sample, a subsample of 500 mg was weighted using a calibrated scale (Mettler Toledo, AT 261 Delta Range, OH, USA), transferred into a glass vial (20 mL headspace vial, Thames Restek, Saunderton, UK) and re-stored directly in a −24 °C freezer until further handling. This amount of sample was chosen to provide an optimum ratio of VOCs to the sample headspace, as validated in a previous sampling method study for pattern-recognition analysis on faecal VOCs using field asymmetric ion mobility spectrometry [32]. For the faecal VOC analysis, the subsamples were shipped on dry ice to the School of Engineering, University of Warwick (Coventry, UK).

### 2.4. Faecal Volatile Organic Compound Analysis

Faecal samples were analysed using gas chromatography (GC) coupled to an ion mobility spectrometer (IMS) (GC-IMS, FlavourSpec^®^, G.A.S., Dortmund, Germany) [33], which was fitted with a CTC PAL autosampler (CTC, Zwingen, Switzerland). GC undertook pre-separation of the mixture of chemicals in the headspace of the faecal sample, and was then detected by a drift-tube IMS. These chemicals were ionized by means of soft chemical-ionization introduced by a low-radiation tritium (H3) source, creating reactant ions. Ionized chemicals then traveled against the flow of an inert drift gas at atmospheric pressure, driven by an electric field. In general, the larger the molecule, the more times they are struck and lose momentum, extending travel time through the tube. Thus, the time taken to traverse the tube is a combination of mass, charge and geometrical structure. The resulting ion current is measured by an electrometer as a function of time. In this study, samples were heated to 80 °C during the 8 min prior to analyses. The GC used a 15 m, SE-54 column (CS Chromatographie, Germany). GC experiments were performed at 40 °C using nitrogen 99.9% (3.5 bar) as the carrier gas and the IMS was performed at 45 °C using nitrogen as the drift gas. Flow rates were set at 150 mL/min (0.364 kPa) (IMS), and at 20 mL/min (34.175 kPa) for 6 min (GC). The G.A.S. FlavourSpec had a resolving power of 75 (defined as the ion drift time divided by the full width at half maximum of the peak—in our case using the reactive ion peak of the background) [34].

### 2.5. Statistical Analysis

Demographics of each group (CD, RCD, HC) were compared using the non-parametric Kruskal–Wallis H test with the addition of the Wilcoxon rank-sum test for continuous data. The Fisher–Freeman—Halton exact test was performed for dichotomous data and Fisher’s exact test for dichotomous data in subanalyses. The statistical tests were performed using IBM SPSS^®^ version 22. Ion mobility spectrometry data was first pre-processed prior to the statistical analyses. Areas that contained chemical information were cropped and a threshold was applied to remove background noise. The data was corrected for instrumental disturbances by baseline realignment. Classification was then undertaken using a 10-fold cross-validated approach, where the data was split into a 90% training set and a 10% test set. Within a fold, the Wilcoxon rank-sum test was used to find the 100 most discriminatory features and these features were then used to train five different classifiers (specifically: sparse logistic regression, random forest, Gaussian process, support vector machine and neural net). These models were then applied to the test set. This process was repeated until every sample had been classified as a test sample and from the resultant classification probabilities, statistical results were calculated.

### 2.6. Ethical Considerations

This study was approved by the local medical ethics committee (METc) of Amsterdam UMC location Vrije Universiteit medical centre (file number 15.368). Written informed consent was obtained from all study participants.

## 3. Results

### 3.1. Baseline Characteristics

A total of 30 patients were included; 13 CD, 7 RCD II, 10 HC. Key symptoms leading to gastroduodenoscopy in the HC group were: retrosternal pain (30%), globus (30%), idiopathic iron deficiency anaemia (20%), reflux (10%) or change in bowel habits (10%). No macroscopic mucosal abnormalities were found in 70% of the cases, and 30% displayed mild forms of gastritis. Baseline characteristics are depicted in Table 1. Age at time of sample collection was highest in the RCD group and lowest in the HC group. The body mass index (BMI) was higher in the HC group. There was no significant difference in the use of a proton pump inhibitor (PPI) or oral antibiotics (OAB) three months prior to inclusion. Seventy-one percent of all RCD patients received anti-inflammatory or immunosuppressive therapy three months prior to sample collection versus 15% of CD patients (*p* = 0.022) and none from the HC (*p* = 0.003) group. Anti-inflammatory therapy in RCD included budesonide in all five, whereas the two CD patients were treated with thiopurines for microscopic colitis. All CD and RCD patients reported adherence to a strict GFD. All HC self-reportedly followed a gluten-containing diet, one HC reported a restricted lactose intake.

### 3.2. Faecal Volatile Organic Compound Analysis

**Figure 1:**
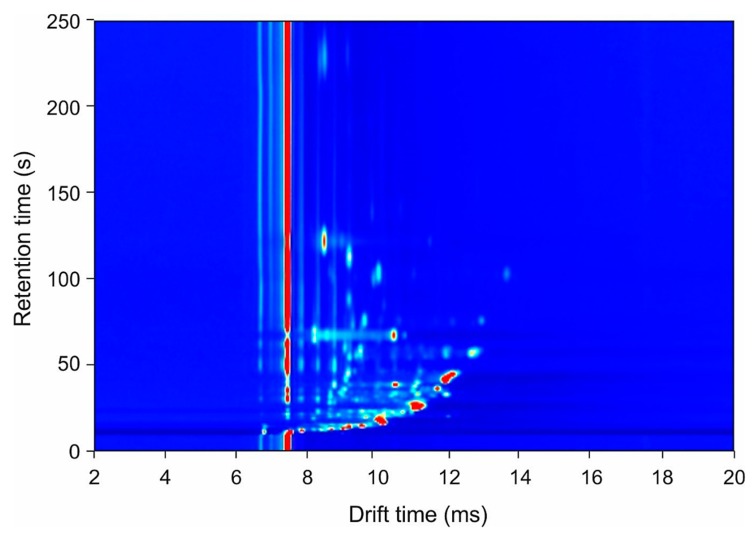
An example output of the gas chromatography–ion mobility spectrometry (GC-IMS) instrument. Depicted is an example output of the GC-IMS (FlavourSpec^®^, G.A.S., Dortmund, Germany) using a refractory coeliac disease sample. The *y*-axis represents retention time from the gas chromatography column, and the *x*-axis represents drift time through the ion mobility spectrometer. Levels of volatile organic compounds in the sample are represented by colour intensity.

For every comparison, the outcomes of the support vector machine classifier are presented. The results of all five classifiers are presented in Appendix A. The results of the faecal VOC measurements by means of GC-IMS are given in Table 2. An example output (response), a chromatogram, of the GC-IMS instrument is depicted in Figure 1. The figure depicts a topographic map of a RCD sample. Each data point is characterised by the retention time in the gas chromatography column. The drift time (milliseconds) and the intensity of the ion current signal is indicated by colour.

**Figure 2:**
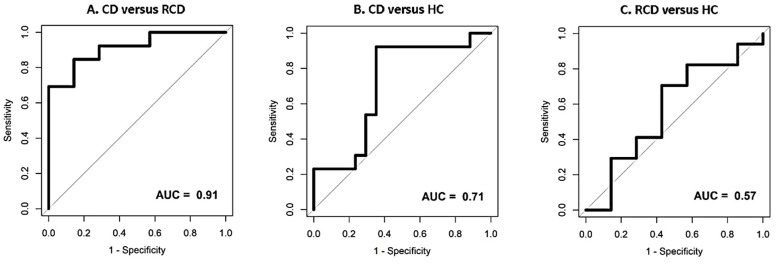
Receiver operator characteristic (ROC) curves for the differentiation between coeliac disease, refractory coeliac disease and healthy controls using faecal volatile organic compounds. (**A**) Coeliac disease versus refractory coeliac disease; (**B**) coeliac disease versus healthy controls; (**C**) refractory coeliac disease versus healthy controls. Figures are generated using the support vector machine based on the 100 most discriminatory features. Abbreviations: AUC, area under the curve; CD, coeliac disease; RCD, refractory coeliac disease; HC, healthy controls.

**Figure 3:**
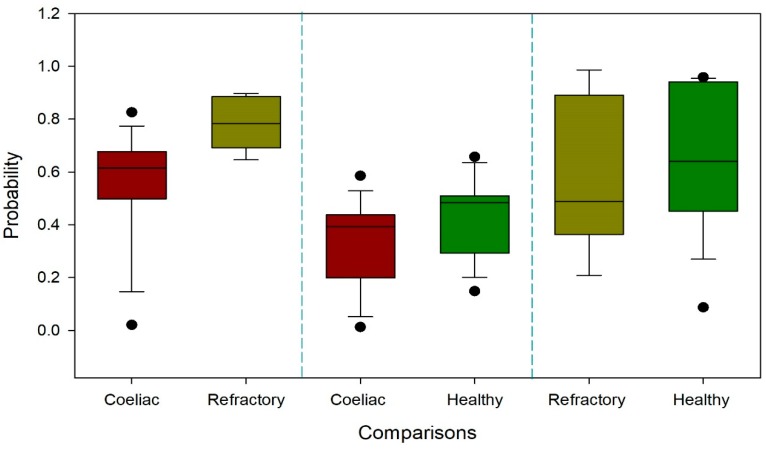
Boxplots represent the probability of the classifier categorizing samples into the correct subgroup. The *x*-axis depicts the subgroups, and the *y*-axis depicts the probability. Boxplots are split into the groups of interest for every comparison, allowing for visualization of the variation within subgroups. Probability is calculated based on the support vector machine classifier.

Using faecal VOC patterns, CD and RCD patients could be discriminated with high accuracy (AUC ± 95% CI: 0.91 (0.79–1), *p* = 0.000). Moderate accuracy was found for the differentiation between CD and HC (AUC ± 95% CI: 0.71 (0.51–0.91), *p*= 0.024). There were no significant differences between the faecal VOC patterns of RCD patients and HC (AUC ± 95% CI: 0.57 (0.29–0.86), *p* = 0.310). Receiver operator characteristic (ROC) curves for the comparisons are depicted in Figure 2. The probability of the support vector machine (SVM) classifier to categorize samples in the correct category is depicted in Figure 3 for all three comparisons, split per group of interest.

## 4. Discussion

This pilot study was the first study to explore the potential of faecal VOCs as a non-invasive biomarker that can discriminate RCD from CD in patients on a GFD. We observed a significant difference in VOC profiles between CD and RCD patients and between CD and HC. We found no significant differences between faecal VOC samples of HC and RCD patients. Di Cagno and colleagues investigated the faecal microbiota and faecal VOCs of children with untreated CD, treated CD and controls, using gas chromatography-mass spectrometry solid-phase micro extraction analysis of VOCs. Not only did they observe differences in microbiome diversity and VOCs between CD and controls, a difference in VOCs was also observed in treated versus untreated CD [30]. In other gastrointestinal diseases, like inflammatory bowel disease, colorectal cancer and multiple infectious diseases, the analysis of faecal VOCs has already been proven to be a promising non-invasive diagnostic tool [26,31,35,36]. In a recent study, faecal VOCs of 497 faecal samples of 281 inflammatory bowel patients and 224 samples of healthy controls were analysed by means of GS-IMS. Inflammatory bowel disease (with and without disease activity) could be discriminated from healthy controls with high accuracy (AUC ± 95% CI: 0.97 (0.94–1.00), *p* = 0.000) [35]. In colorectal cancer and advanced adenomas, a high diagnostic value for the analyses of VOCs is observed [31]. These results strengthen the promise held by the analysis of faecal VOCs as a non-invasive biomarker in gastrointestinal diseases (e.g., CD and RCD).

Since this study was a pilot study, the number of patients included was limited. We hypothesize that the inability to discriminate RCD from HC might have been attributable to the small sample size. The heterogeneity of VOC profiles of both groups is indirectly demonstrated by the length of the boxplots in Figure 3. It could be hypothesized that the individual heterogeneity of VOCs within these groups has made it more difficult to pick up group-specific differences in VOCs between HC and RCD, consequently impeding proper validation of the yielded training data. As a result, an eligible cut off for the determination of the sensitivity and specificity could not be produced for the comparison of RCD and HC, nor was this appropriately possible for CD and HC. This might be caused by the relatively increased influence of individual bias factors due to the small size. Collection and, in most cases, transportation was done by the participant, limiting control over the count of freeze-thaw cycles the sample went through. Also, in the process of sample collection by the participant, there might be differences in, for instance, the amount of water collected with the stool sample. These variables have both been described as influential factors in analysing faecal VOCs by means of VOC pattern recognition with an electronic nose [27]. However, we do not expect them to have played a large role in this study since we used CG-IMS which is a more sensitive technique as it permits distinction on a molecular level. Another strength of this study was that bias by other gastric diseases was limited by including an endoscopy-controlled HC group. It has been shown that diet, smoking and medication can alter the composition of the microbiome [37,38,39]. The VOC profile is also altered by smoking and it is plausible that this may hold for diet and medication as well [40]. There were no major differences between groups concerning the abovementioned variables, except for the budesonide therapy. Five RCD patients received budesonide therapy versus none of the healthy controls (*p =* 0.003). This could have been of influence on the VOC profile. Based on VOC pattern recognition RCD was discriminated from CD with a sensitivity of 0.85 and a specificity of 0.86. These results hold great promise for the future and require validation in a larger cohort. We hypothesize that a larger sample size will temper the effect of personal bias on the heterogeneity, enabling machine learning to reduce this background noise.

Currently, a (repeat) gastroduodenoscopy with the histological examination of duodenal biopsies and flow cytometric analysis of freshly isolated duodenal lymphocytes are necessary to confirm RCD [12]. The procedure itself as well as the histopathological examination and flow-cytometry are costly and time consuming. More importantly, a gastroduodenoscopy is an invasive procedure, leading to discomfort in already unwell patients, and possibly leading to complications (e.g., perforation or bleeding) [14].

Future research focusing on faecal VOC profiles as novel non-invasive biomarker for RCD detection and monitoring may use chemical analytical platforms as gas chromatography–mass spectrometry (GC-MS) or pattern-recognition techniques like GC-IMS. While the first analytical platform has the potential to identify the specific metabolites differentiating between groups of interest, it is an expensive and time-consuming method that requires specialized personnel. The use of pattern recognition is favourable for clinical use, as it allows for fast, relatively low-cost and high-throughput analysis. Ideally, should differences in faecal VOC profiles of CD, RCD and HC be validated in a larger cohort, a disease specific faecal VOC print may be calculated, making VOC analysis a practical and cost-effective method for the evaluation of the discrimination between RCD and normal CD.

## 5. Conclusions

In conclusion, we observed significant differences in faecal VOC profiles between CD and RCD patients using GC-IMS, and to a lesser extent between CD and HC, but not between RCD and HC. Our study outcomes imply that faecal VOC analysis may hold potential as a non-invasive biomarker for the discrimination between CD and RCD patients. Future research should focus on the validation of these findings in a larger cohort. The calculation of a specific faecal VOC print could make VOC analysis feasible as a more patient friendly and cost-effective diagnostic marker for RCD.

## Figures and Tables

**Table 1:** Baseline characteristics.

	Coeliac Disease (*n* = 13)	Refractory Coeliac Disease (*n* = 7)	Healthy Controls (*n* = 10)	*p*-Value ALL	*p*-Value CD vs. HC	*p*-Value RCD vs HC	*p*-Value CD vs. RCD
Sex, female (*n*, [%])	10 [76%]	3 [42%]	6 [60%]	0.314	0.650	0.637	0.174
Age (median [IQR])	69 [44–78]	78 [76–80]	59 [46–71]	0.025 *	0.085	0.001 **	0.085
BMI (median [IQR])	23 [20–28]	23 [20–24]	28 [26–33]	0.027 *	0.588	0.010 *	0.588
BSS (median [IQR])	4 [1.5–4.5]	4 [3.0–6.0]	3 [3.0–5.0]	0.628	0.371	0.470	0.371
Currently Smoking (*n*, [%])	1 [7.7%]	0	1 [10%]	1.000	1.000	1.000	1.000
Proton Pump Inhibitors (*n*, [%])	5 [39%]	6 [86%]	4 [40%]	0.130	1.000	0.134	0.070
Antibiotics (*n*, [%])	4 [31%]	2 [29%]	0	0.121	0.104	0.154	1.000
Immunosuppressive therapy	2 [15%] ^$^	5 [71%] ^#^	0	0.002 *	0.486	0.003 **	0.022 *

CD: coeliac disease, HC: healthy control, RCD: refractory coeliac disease, BMI: body mass index, BSS: Bristol stool scale, IQR: interquartile range antibiotics, proton pump inhibitors and immunosuppressive medication used in the last 3 months, ^$^: thiopurines, ^#^: budesonide. * *p*-value < 0.050, ** *p*-value < 0.010.

**Table 2:** Differences in faecal volatile organic compounds between patients with coeliac disease, refractory coeliac disease and healthy controls.

Comparison	AUC (95% CI)	Sensitivity	Specificity	PPV	NPV	*P*-Value
Coeliac disease vs. refractory coeliac disease	0.91 (0.79–1)	0.85	0.86	0.92	0.75	0.000
Coeliac disease vs. healthy controls	0.71 (0.51–0.91)	0.92	0.65	0.67	0.92	0.024
Refractory coeliac disease vs. healthy controls	0.57 (0.29–0.86)	0.71	0.57	0.80	0.44	0.310

Outcomes obtained using support vector machine (SVM) analyses based on the 100 most discriminating features. Abbreviations: AUC, area under the curve; CI, confidence interval; PPV, positive predictive value; NPV, negative predictive value; CD, coeliac disease; RCD, refractory coeliac disease; HC, healthy controls.

## Appendix A

Coeliac vs. refractory 100 features
Sparse logistic regression (ROC)
AUC = 0.8 (0.6–1)
sensitivity = 0.62 (0.32–0.86)
specificity = 1 (0.59–1)
PPV = 1
NPV = 0.58
*p*-Value = 0.013
Random forest (ROC)
AUC = 0.81 (0.62–1)
sensitivity = 0.62 (0.32–0.86)
specificity = 1 (0.59–1)
PPV = 1
NPV = 0.58
*p*-Value = 0.011
Gaussian process (ROC)
AUC = 0.92 (0.81–1)
sensitivity = 0.77 (0.46–0.95)
specificity = 1 (0.59–1)
PPV = 1
NPV = 0.7
*p*-Value = 0.000
Support vector machine (ROC)
AUC = 0.91 (0.79–1)
sensitivity = 0.85 (0.55–0.98)
specificity = 0.86 (0.42–1)
PPV = 0.92
NPV = 0.75
*p*-Value = 0.000
Neural net (ROC)
AUC = 0.76 (0.54–0.98)
sensitivity = 0.54 (0.25–0.81)
specificity = 1 (0.59–1)
PPV = 1
NPV = 0.54
*p*-Value = 0.028
Celiac vs. healthy 100 features
Sparse logistic regression (ROC)
AUC = 0.57 (0.34–0.79)
sensitivity = 0.38 (0.14–0.68)
specificity = 0.94 (0.71–1)
PPV = 0.83
NPV = 0.67
*p*-Value = 0.268
Random forest (ROC)
AUC = 0.51 (0.28 - 0.75)
sensitivity = 0.38 (0.14 - 0.68)
specificity = 0.88 (0.64 - 0.99)
PPV = 0.71
NPV = 0.65
*p*-Value = 0.442
Gaussian process (ROC)
AUC = 0.5 (0.26–0.74)
sensitivity = 0.23 (0.05–0.54)
specificity = 1 (0.8–1)
PPV = 1
NPV = 0.63
*p*-Value = 0.5
Support vector machine (ROC)
AUC = 0.71 (0.51–0.91)
sensitivity = 0.92 (0.64–1)
specificity = 0.65 (0.38–0.86)
PPV = 0.67
NPV = 0.92
*p*-Value = 0.024
Neural net (ROC)
AUC = 0.62 (0.39–0.85)
sensitivity = 0.54 (0.25–0.81)
specificity = 0.88 (0.64–0.99)
PPV = 0.78
NPV = 0.71
*p*-Value = 0.129
Refractory vs. healthy 100 features
Sparse logistic regression (ROC)
AUC = 0.42 (0.13–0.71)
sensitivity = 0.82 (0.57–0.96)
specificity = 0.29 (0.037–0.71)
PPV = 0.74
NPV = 0.4
*p*-Value = 0.284
Random forest (ROC)
AUC = 0.42 (0.14–0.7)
sensitivity = 0.59 (0.33–0.82)
specificity = 0.57 (0.18–0.9)
PPV = 0.77
NPV = 0.36
*p*-Value = 0.2834
Gaussian process (ROC)
AUC = 0.59 (0.29–0.89)
sensitivity = 0.82 (0.57–0.96)
specificity = 0.57 (0.18–0.9)
PPV = 0.82
NPV = 0.57
*p*-Value = 0.267
Support vector machine (ROC)
AUC = 0.57 (0.29–0.86)
sensitivity = 0.71 (0.44–0.9)
specificity = 0.57 (0.18–0.9)
PPV = 0.8
NPV = 0.44
*p*-Value = 0.310
Neural net (ROC)
AUC = 0.6 (0.35–0.84)
sensitivity = 0.35 (0.14–0.62)
specificity = 1 (0.59–1)
PPV = 1
NPV = 0.39
*p*-Value = 0.772
